# Sequence-dependent catalytic regulation of the SpoIIIE motor activity ensures directionality of DNA translocation

**DOI:** 10.1038/s41598-018-23400-8

**Published:** 2018-03-27

**Authors:** Osvaldo Chara, Augusto Borges, Pierre-Emmanuel Milhiet, Marcelo Nöllmann, Diego I. Cattoni

**Affiliations:** 1 Systems Biology Group (SysBio), Institute of Physics of Liquids and Biological Systems (IFLySIB), National Scientific and Technical Research Council (CONICET), University of La Plata (UNLP), Calle 59 No 789 (1900), La Plata, Argentina; 2 Center for Information Services and High Performance Computing (ZIH), Technische Universität Dresden (TUD), Nöthnitzerstr 46, 01069 Dresden, Germany; 3 Centre de Biochimie Structurale, CNRS UMR5048, INSERM U1054, Université de Montpellier, 29 rue de Navacelles, 34090 Montpellier, France

## Abstract

Transport of cellular cargo by molecular motors requires directionality to ensure proper biological functioning. During sporulation in Bacillus subtilis, directionality of chromosome transport is mediated by the interaction between the membrane-bound DNA translocase SpoIIIE and specific octameric sequences (SRS). Whether SRS regulate directionality by recruiting and orienting SpoIIIE or by simply catalyzing its translocation activity is still unclear. By using atomic force microscopy and single-round fast kinetics translocation assays we determined the localization and dynamics of diffusing and translocating SpoIIIE complexes on DNA with or without SRS. Our findings combined with mathematical modelling revealed that SpoIIIE directionality is not regulated by protein recruitment to SRS but rather by a fine-tuned balance among the rates governing SpoIIIE-DNA interactions and the probability of starting translocation modulated by SRS. Additionally, we found that SpoIIIE can start translocation from non-specific DNA, providing an alternative active search mechanism for SRS located beyond the exploratory length defined by 1D diffusion. These findings are relevant *in vivo* in the context of chromosome transport through an open channel, where SpoIIIE can rapidly explore DNA while directionality is modulated by the probability of translocation initiation upon interaction with SRS versus non-specific DNA.

## Introduction

Bacterial DNA pumps are responsible for the intra and intercellular transfer of genetic material across membranes during sporulation, cell division and conjugation^1^. SpoIIIE is responsible for the directional translocation of double-stranded DNA during sporulation and cell division in *Bacillus subtilis* (*B*. *subtilis*)^2–6^. SpoIIIE is composed of an N-terminal transmembrane domain involved in septal localization^4^, an unstructured linker and a C-terminal motor domain that assembles as a hexameric ring^7^. The cytoplasmic motor domain of SpoIIIE can be subdivided into three separate subdomains: the α and β subdomains that contain the core ATPase machinery ensuring DNA translocation and the γ subdomain that recognizes a tandem of 8 base pair repeats (termed SRS for SpoIIIE Recognition Sequence) in an orientation-specific manner^6^. Similarly to other Rec-A like NTPases, SpoIIIE preferentially tracks the 5′ → 3′ strand in the direction of translocation^8,9^.

The detailed molecular mechanism by which SRS regulates motor directionality is still a matter of debate^10^. It has been shown that SpoIIIE has higher affinity for SRS than for non-specific DNA and that SRS stimulates its ATPase activity^11,12^, suggesting that SRS could either regulate directionality by recruiting and orienting SpoIIIE hexamers prior to engaging translocation or by simply triggering SpoIIIE translocation. Predictions based solely on ATPase measurements were recently challenged by a study showing that SpoIIIE ATPase and translocation activities can be decoupled by mutating specific residues^13^. Hence, the distinction between these two mechanisms (recruitment and orienting *vs*. catalytic triggering) requires direct observation of SpoIIIE-DNA complexes and discrimination between the translocating state when complexes are interacting with non-specific DNA or with SRS.

Here, we study how sequence recognition regulates the directionality and translocation activity of SpoIIIE by combining single molecule imaging with single round translocation measurements and mathematical modelling. We directly observed and quantified the number of diffusing and translocating complexes on DNA with and without SRS by atomic force microscopy (AFM) and further analyzed their dynamics by single-round translocation kinetics measurements (triplex displacement assay). To interpret our experimental results, we developed a mathematical model accounting for all the biochemical processes describing the interaction between SpoIIIE and DNA (*i*.*e*., association, dissociation, 1D diffusion, ATP activation and translocation). We show that, in contrast to predictions from previous models^12^, SpoIIIE can actively translocate on DNA independently of the presence of specific sequences, and that SRS modulates the translocation activity of SpoIIIE. Our results strongly suggest that rather than recruiting and orienting the non-active protein, SRS sequences regulate directionality by modulating  the translocation activity of SpoIIIE.

## Results

### SpoIIIE can efficiently couple ATP hydrolysis to translocation independently of the presence of SRS sequences

**Figure 1:**
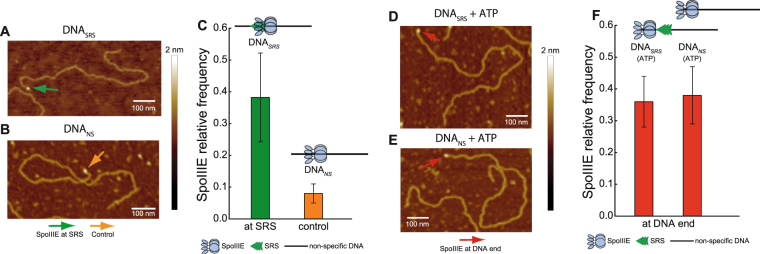
SpoIIIE can actively translocate and reach DNA ends even in the absence of SRS sequences. (**A**,**B**) AFM micrographs of SpoIIIE bound to SRS (DNA_*SRS*_) and to non-specific DNA (DNA_*NS*_) in the absence of ATP. Arrows indicate SpoIIIE complexes bound to SRS (green arrow in panel A) and to equivalent position replaced by non-specific DNA as a control (orange arrow in panel B). (**C**) Relative frequency of SpoIIIE binding to SRS (DNA_*SRS*_) or to the equivalent position replaced by non-specific DNA (DNA_*NS*_, control). The position of SpoIIIE complexes in DNA was determined using a semi-automated algorithm and the relative frequency of SpoIIIE localization in bins of 50 nm was calculated (see Fig. S1A,B, Material and Methods and Supplementary Information). Error bars were obtained from the error propagation of Eq. 1 (n = 35). Upper scheme represents SpoIIIE bound to SRS and to the equivalent position replaced by non-specific DNA. (**D**,**E**) AFM micrographs of SpoIIIE complexes with DNA_*SRS*_ and DNA_*NS*_ substrates in the presence of ATP. Red arrows indicate SpoIIIE complexes that have reached the DNA ends by translocation. (**F**) Relative frequency of SpoIIIE reaching DNA ends by translocation in substrates with (DNA_*SRS*_) or without (DNA_*NS*_) SRS. The position of SpoIIIE complexes in DNA was determined as described in panel C (see Fig. S1C,D, n = 40). Upper scheme represents SpoIIIE reaching the DNA ends for substrates with or without SRS. Relative frequencies depicted in panel C were recalculated from previously published data^11^.

Different studies agree that SpoIIIE activity is stimulated by SRS^11,12^, however the capacity of SpoIIIE to efficiently couple ATP hydrolysis to translocation in absence of SRS remains unclear. Based on ATPase activity measurements, it has been proposed that SpoIIIE activity is inhibited in absence of SRS sequences^12^. However, biochemical and single molecule studies reported the capacity of SpoIIIE to hydrolyze ATP and to translocate when interacting with non-specific DNA^6,11^. ATPase activity of the motor allows to infer translocation, but may not be a definitive proof as the enzyme could be performing futile cycles of ATP hydrolysis (*i.e.* not converting the chemical energy into mechanical movement) or ATP hydrolysis may go undetected depending on the methodology employed. On the other hand, single molecule studies do not allow to simultaneously quantify ATP hydrolysis and translocation to conclude unambiguously on the efficiency of the mechanochemical transduction. To directly test the ability of SpoIIIE to couple ATP hydrolysis to translocation independently of the presence of SRS, we performed AFM imaging of SpoIIIE incubated with linearized DNA substrates without or with SRS sequences oriented towards the nearest DNA end (DNA_*NS*_ and DNA_*SRS*_ respectively) in the presence and absence of ATP. First we sought to evaluate the capacity of SpoIIIE to hydrolyze ATP when incubated with either substrate. Interestingly, SpoIIIE displayed equivalent specific ATPase activity in both DNA_*SRS*_ and DNA_*NS*_ (Fig. S1E). When evaluating spatial localization of SpoIIIE in DNA, in absence of ATP 38% of the detected SpoIIIE was bound to SRS in DNA_*SRS*_ (Figs 1A,C and S1A) whereas less than 10% of SpoIIIE located to the equivalent position in DNA_*NS*_ (Figs 1B,C and S1B). Strikingly, when adding ATP SpoIIIE preferentially localized at the DNA ends (~40%) independently of the presence of SRS (Figs 1D–F and S1C,D). These results indicate that SpoIIIE can explore the DNA without using the energy provided by ATP hydrolysis to reach SRS while becoming activated and translocating DNA with similar efficiencies independently of the presence of SRS.

**Figure 2:**
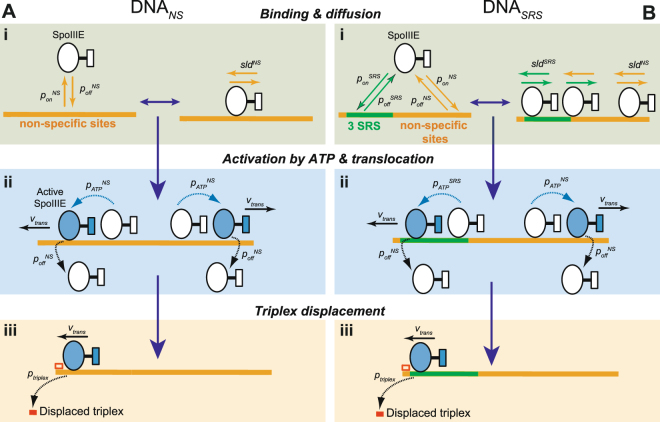
Scheme of the mathematical model encoding the dynamics of SpoIIIE/DNA interaction. (**A**,**B**) In the model SpoIIIE can interact with non-specific DNA (DNA_*NS*_, A) and SRS containing DNA (DNA_*SRS*_, B). The dynamics of non-active SpoIIIE (i) were modelled as a Markov process controlled by the SpoIIIE binding/unbinding probabilities (*p*_*on*_/*p*_*off*_) to/from non-specific or SRS regions of DNA (orange and green solid lines respectively). Once bound to DNA, SpoIIIE can undergo 1D diffusion defined by the sliding length *sld*. In the presence of ATP (ii), SpoIIIE can become active with a probability *p*_*ATP*_ and translocate at constant velocity (*v*_*trans*_). Probabilities of binding, unbinding, sliding lengths and activation by ATP could be set to distinct values depending on the DNA substrate. When the triplex was present (iii), translocating SpoIIIE can displace the triplex when reaching the corresponding DNA end with probability *p*_*triplex*_. See Material and Methods and Supplementary Information for additional details.

To further understand the interaction of SpoIIIE with SRS and non-specific DNA as well as the regulation of its translocation activity, a mathematical model describing the binding and diffusing properties of SpoIIIE^11^ was extended to include DNA translocation. The model mimics the two experimental scenarios for DNA with or without SRS sequences (Fig. 2A,B). Briefly, SpoIIIE is able to interact non-deterministically with SRS and non-specific DNA sequences with distinct association and dissociation probabilities *per* time step *p*_*on*_^*SRS*^, *p*_*on*_^*NS*^, *p*_*off*_^*SRS*^ and *p*_*off*_^*NS*^. When bound to DNA, SpoIIIE can undergo 1D diffusion or sliding, with sliding lengths *sld*^*SRS*^ and *sld*^*NS*^ for SRS and non-specific DNA, respectively (Fig. 2A–Bi), and/or become activated by ATP with a probability *p*_*ATP*_ to start translocating with uniform velocity *v*_*trans*_ (Fig. 2A–Bii). Direction of translocation with probability *p*_*dir*_ = 0.5 in either direction (*i*.*e*., 50% chance for each direction) is defined when SpoIIIE is activated and remains constant until the motor dissociates. While translocating SpoIIIE can dissociate from DNA with *p*_*off*_^*NS*^ probability (see Materials and Methods and Supplementary Information for a full description of the model). For all conditions studied, distributions of SpoIIIE were collected after the system reached equilibrium (~2000 Monte Carlo steps) during 1000 Monte Carlo steps (Fig. S4).

**Figure 3:**
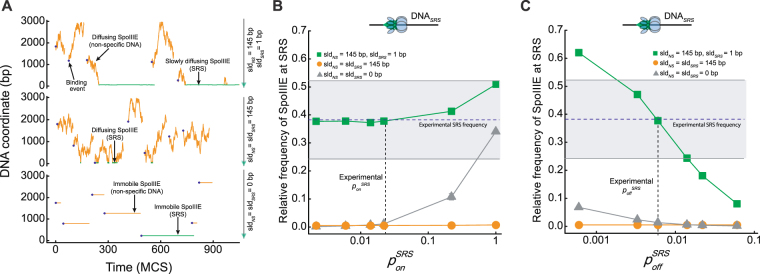
Model predicted dynamics and distributions of SpoIIIE in DNA_*SRS*_ in the absence of ATP. (**A**) Representative trajectories of SpoIIIE interacting with *DNA*_*SRS*_ in the absence of ATP. Each panel represents a DNA molecule where the sliding properties of SpoIIIE when interacting with SRS and non-specific DNA were varied with parameter values shown on the right of each panel. The rest of the parameters were set to the optimal values depicted in Table 1. Horizontal and vertical axis represent time evolution in Monte Carlo steps (MCS) and DNA coordinate (in base pairs, bp), respectively. SpoIIIE binding events are shown by solid blue hexagons and trajectories are shown in orange and green when interacting with non-specific DNA and SRS, respectively. Scheme on the right depicts non-specific DNA regions (solid black line) and SRS sites (semi-transparent green triangles). (**B**,**C**) The model-predicted relative frequency of SpoIIIE at SRS sequences in the absence of ATP was evaluated as function of the SpoIIIE/SRS association and dissociation probabilities (*p*_*on*_^*SR*S^ and *p*_*off*_^*SR*S^ for panels B and C, respectively) one order of magnitude above and below the values reproducing AFM distributions while the rest of the parameter values were set to the values described in Table 1. Simulations were performed in the absence of 1D diffusion (grey triangles), including homogeneous (orange circles) and anomalous (green squares) sliding between non-specific DNA and SRS with values depicted in the inset of the figure. Solid lines connecting dots are only a guide to the eye. Blue dotted line and grey shaded area indicate the mean experimental relative frequency of SpoIIIE binding at SRS and standard deviation respectively as obtained from Fig. 1C. Dotted black vertical line indicates the association and dissociation probability values defined from experimental data (see Methods and Supplementary Information). Upper scheme represents SpoIIIE bound to SRS.

First, we assessed the model in the absence of translocation and evaluated the relative frequency of SpoIIIE binding to SRS. Experimentally it has been shown that SpoIIIE associates with identical rates to SRS and non-specific DNA, whereas dissociation from SRS is ~3 times slower^11^. The model parameters were set to values emulating these experimental binding rates and different sliding lengths of SpoIIIE when interacting with non-specific DNA or SRS were explored (Fig. S2). AFM distributions for DNA_SRS_ were recovered when SpoIIIE explored large regions of non-specific DNA (*sld*^*NS*^ = 145 bp) whereas it was almost immobile when bound to SRS (*sld*^*SRS*^ = 1 bp, Fig. 3A upper panel and S2 and Movie M1). Equivalent results were obtained when employing anomalous 3D hopping as DNA exploring mechanism (unpublished data). To evaluate the robustness of the model, the binding and diffusion parameters were varied at least one order of magnitude above and below the optimal values (Fig. 3B,C and Table 1). When 1D diffusion was absent or equivalent between non-specific DNA and SRS, experimental distributions were not recovered for the large majority of the parameter values (Fig. 3B,C, see Fig. S2 for discussion of these results). Detailed observation of single molecule trajectories revealed that SpoIIIE either did not remain bound to SRS or the frequency of interaction with SRS by direct binding was extremely low in this conditions (Fig. 3A middle and lower panels respectively). When the ratio sld_*NS*_/sld_*SRS*_ was set at 145, the model reproduced the AFM obtained distributions for a large set of association probabilities, whereas in similar conditions only a narrow set of dissociation probabilities values reproduced the localization of SpoIIIE to SRS (Fig. 3B,C). These results indicate the critical role of anomalous diffusion (sld_*NS*_ >> sld_*SRS*_) and SRS dissociation rate (*p*_*off*_^*SRS*^ < *p*_*off*_^*NS*^) in regulating DNA exploration and stable binding to ensure SRS recognition.

**Table 1:** Model parameters values satisfying experimental results.

Parameter	*DNA*_*NS*_	*DNA*_*SRS*_
*p*_*on*_^*SRS*^	—	0.022
*p*_*on*_^*NS*^	0.022	0.022
*p*_*off*_^*SRS*^	—	0.006
*p*_*off*_^*NS*^	0.014	0.014
*sld* ^*SRS*^	—	1.0 ± 0.25 (bp)
*sld* ^*NS*^	145 ± 36 (bp)	145 ± 36 (bp)
*p*_*ATP*_^*SRS*^	—	≥0.4
*p*_*ATP*_^*NS*^	0.012	0.012
*v*_*trans*_	5 (kb/s)	5 (kb/s)

**Figure 4:**
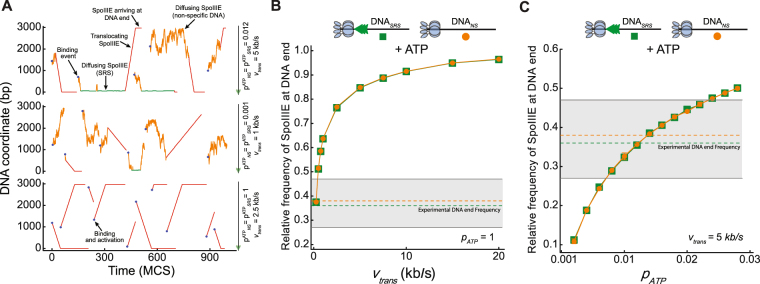
Model predicted dynamics and distributions of SpoIIIE in DNA_*SRS*_ and DNA_*NS*_ in the presence of ATP. (**A**) Representative trajectories of SpoIIIE when interacting with *DNA*_*SRS*_ in the presence of ATP. Each panel represents a DNA molecule where the activation probability (*p*_*ATP*_) and translocating velocity (*v*_*trans*_) were varied with values shown on the right of each panel. The rest of the parameters were set at the values depicted in Table 1. Horizontal and vertical axis represent time evolution in Monte Carlo steps (MCS) and DNA coordinates (in base pairs, bp), respectively. Color code from Fig. 3A is conserved and translocating proteins are depicted in red. Translocation velocity was reduced 10 times with respect to the value depicted on the right solely for representation purposes. Scheme on the right depicts non-specific DNA regions (solid black line) and SRS sites (semi-transparent green triangles). (**B**,**C**) Model-predicted relative frequency of SpoIIIE reaching DNA ends for DNA_*SRS*_ (green squares) and DNA_*NS*_ (orange circles) as a function of translocation velocity with ATP activation probability *p*_*ATP*_ = 1 (**B**) and as a function of *p*_*ATP*_ for different translocation velocities (**C**). Solid lines connecting dots are only a guide to the eye. Orange and green dashed lines and grey shadow area indicate the experimental mean relative frequency and standard deviation respectively for both substrates in the presence of ATP. All other parameter values were set at the values depicted in Table 1. Upper scheme represents SpoIIIE reaching the DNA ends for substrates with or without SRS.

Next, we evaluated the localization of SpoIIIE when incubated with DNA_*SRS*_ and DNA_*NS*_ in the presence of ATP and quantified the relative frequency of proteins reaching the DNA ends. First, using the previously validated parameter values (Figs 3B,C and S2 and Table 1), we tested the simplest case in which all proteins became instantly active after binding to DNA (*p*_*ATP*_ = 1) for a large range of translocating velocities. Under these parametric settings proteins do not explore DNA by 1D diffusion (Fig. 4A lower panel) and simulations showed that the relative number of SpoIIIE located at the DNA ends hyperbolically increased with translocation velocities to values largely exceeding AFM observations for both DNA_*NS*_ and DNA_*SRS*_ (Fig. 4B). Notably, the translocation velocities reproducing experimental SpoIIIE distributions on DNA were below 0.1 kb.s^−1^ (Fig. 4B), a value far below the reported translocation velocities in equivalent experimental conditions (4 to 7 kb.s^−1^ at 20 **°**C^6^). We reasoned that the model did not reproduce experimental DNA distributions of SpoIIIE due to the extreme conditions imposed: instantaneous activation of DNA-bound motors is biologically unlikely. When reducing the probability of activation by ATP, SpoIIIE was able to randomly bind, explore large regions of non-specific DNA and become stochastically activated (Fig. 4A upper and middle panels). Remarkably, simulations showed that the model recovers the experimental localization of SpoIIIE at DNA ends when *p*_*ATP*_ ranges between 0.012 and 0.014 (Fig. 4C) for both DNA_*SRS*_ and DNA_*NS*_ and with translocation velocities in agreement with previously reported values (Fig. 4A upper panel, 4C and S3A,B and Movies M2 and M4). When employing lower translocation velocities and/or activation probabilities the number of SpoIIIE reaching the DNA ends was significantly lower than experimentally predicted since SpoIIIE either dissociates before becoming active or during translocation (Fig. 4A middle panel and 4C). To further verify the biological significance of the optimal parameter values predicted by our modelling, we extracted from the simulations the mean time employed by SpoIIIE to explore DNA and reach SRS sequences as well as the time SpoIIIE remains bound to DNA in a single translocation event (Fig. S5). Our results indicate that SpoIIIE explores DNA for 2.8 ± 24 seconds before targeting SRS and it is able to translocate for 5.2 ± 6.8 seconds *per* single round. These values are in very good agreement with *in vivo* measurements for transcription factors targeting of specific sites^14,15^ and, when assuming a translocation velocity of ~5 kb/s, with measured procesivities for SpoIIIE homologues (5 to 20 kb,^9,16^). All in all, our modelling results show that, in the absence of ATP, the preferential localization of SpoIIIE at SRS is satisfactorily reproduced by a very narrow range of parameter values that is fully compatible with previously published experimental data, indicating that efficient recognition of SRS relies on both anomalous diffusion allowing SpoIIIE to explore large regions of DNA and low dissociation constant from SRS to ensure stable binding. On the other hand, when ATP is present, SpoIIIE reached the DNA ends when the probabilities of activation by ATP were similar to the binding/unbinding probabilities and independently of the presence of SRS. Then, if the presence of SRS is not essential for SpoIIIE to initiate translocation and it does not affect the distributions of proteins when ATP is available, what is the exact mechanism by which SRS establishes directionality of the SpoIIIE motor?

### SpoIIIE translocation directionality is catalytically regulated by SRS

**Figure 5:**
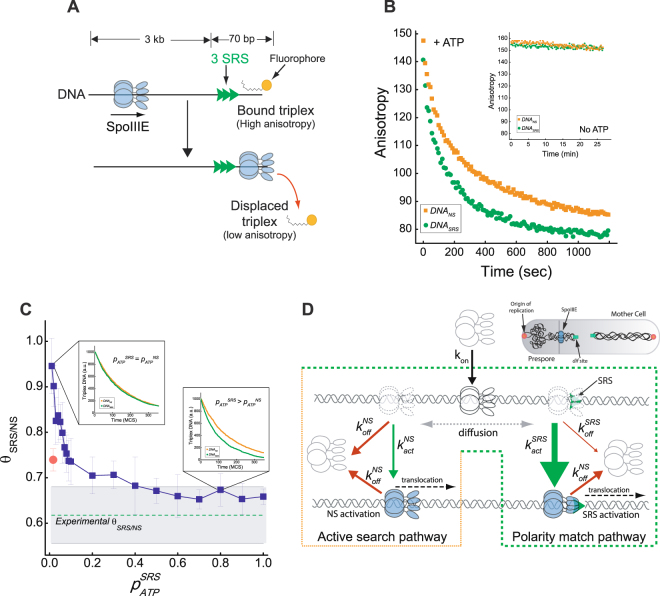
SRS enhances ATP-dependent translocation activity of SpoIIIE. (**A**) Scheme representing the triplex displacement experimental design. (**B**) Time course of triplex displacement by SpoIIIE for substrates with (green circles) or without (orange squares) SRS sequences in the presence of ATP. Inset shows that in the absence of ATP, SpoIIIE is unable to displace the triplex. (**C**) Effect of SRS in triplex displacement rates quantified as the ratio between the area under the kinetic traces for DNA_*SRS*_ and DNA_*NS*_ (*θ*_*SRS/NS*_). Blue squares and error bars indicate mean and standard deviation from the simulation results by varying *p*_*ATP*_^*SRS*^ while maintaining *p*_*ATP*_^*NS*^ constant at 0.012. Red circle depicts the result expected for the ‘recruitment and orient’ directionality model (see main text). Green dotted line and grey shaded area indicate the mean and standard deviation of *θ*_*SRS/NS*_ obtained from the experimental results shown in panel B (*n* = 3). Solid blue lines connecting squares are only a guide to the eye. Insets depict the time courses for triplex displacement obtained from simulations when *p*_*ATP*_^*SRS*^ = *p*_*ATP*_^*NS*^ (left) and *p*_*ATP*_^*SRS*^ = 0.8 (right). Parameter values for simulations are depicted in Table 1. (**D**) SpoIIIE *in vivo* directionality mechanism. Free, bound, diffusing and active SpoIIIE are represented in grey, black, dotted grey and filled light blue respectively. Dotted green and orange contour lines represent the two alternative pathways for SpoIIIE directionality regulation. Upon binding (black arrow) SpoIIE can explore DNA by diffusion (dotted grey arrow) and/or dissociate (red arrows) and/or become active (green arrows). Once active SpoIIIE can translocate (dashed black arrows). Thickness of red and green arrows is proportional to the rate/probability values obtained from experimental and modelling optimized parameter values when SpoIIIE is interacting with non-specific or SRS sequences. Upper scheme depicts a B. subtilis cell in the initial stages of sporulation. oriC regions (red circles) move towards the cell poles and after asymmetric division SpoIIIE (blue circles) is recruited to the septum to transport two-thirds of the chromosome into the forespore.

To assess if protein translocation activity is regulated by SRS, we implemented a fast kinetics assay with temporal resolution on the seconds scale that allows for monitoring single-round cycles of the enzyme arrival at the DNA end proximal to SRS^6^. The substrates were identical to those employed for AFM measurements but contained a fluorescently labelled triplex-forming oligonucleotide inserted at the 5′ end (Fig. 5A). Displacement of triplex by SpoIIIE was monitored in real time by following the changes in fluorescence anisotropy of the TAMRA-labelled triplex (Fig. 5A,B).

In presence of SpoIIIE but no nucleotide, triplex anisotropy was constant for more than 25 minutes (Fig. 5B inset), indicating that the triplex was stably bound and that diffusing SpoIIIE did not displace them. In contrast, in the presence of ATP and SpoIIIE, the triplex anisotropy rapidly decreased with time (Fig. 5B), indicating that ATP-fueled DNA translocation by SpoIIIE leads to triplex displacement. Interestingly, the kinetics of triplex displacement by SpoIIIE were faster in the substrates containing SRS (Fig. 5B). The differences in kinetics between substrates was quantified from the ratio between the areas under the kinetic traces for DNA_*SRS*_ and DNA_*NS*_ (*θ*_*SRS/NS*_ = 0.62 ± 0.06), and showed that SRS accelerates by ~60% the triplex displacement rate. *In vivo* and *in vitro* SpoIIIE is capable of removing from DNA tightly bound complexes such as RNA polymerase and transcription factors^17^. Moreover additional simulations confirmed the capacity of SpoIIIE to reach DNA ends of both substrates in equivalent manner independently of the presence of the triplex (Fig. S6). Thus, the observed differences in the displacement rates should arise from changes in the intrinsic activity of SpoIIIE.

To evaluate the mechanism responsible for accelerated triplex displacement in DNA_*SRS*_, the model was extended to include a triplex at the 5′ DNA end (Fig. 2A–Biii). Kinetic traces in the presence and absence of SRS were simulated and the ratio of the area under the curves (*θ*_*SRS/NS*_) was calculated as for the experimental conditions. Initially, the optimal parameter values obtained in the previous section were employed (*v*_*trans*_ = 5 kb s^−1^ and *p*_*ATP*_ = 0.012, see Table 1). In these conditions, the rates of triplex displacements for substrates with or without SRS were almost equivalent (*θ*_*SRS/NS*_ = 0.95 ± 0.06, Fig. 5C left inset), indicating that the higher affinity of SpoIIIE for SRS is not enough to explain the faster rate of displacement in the presence of SRS. To further test the if protein orientation by SRS is an essential element of the directionality regulation, the directionality parameter (*p*_*dir*_) was varied to modulate the probability of SpoIIIE to translocate in a given direction when activated by SRS. When setting the probability of translocating towards the triplex to a 100%, the model was still unable to reproduce the acceleration observed in triplex displacement when SRS is present (*θ*_*SRS/NS*_ = 0.74 ± 0.02, Fig. 5C red dot). These results indicate that the recruitment of SpoIIIE to SRS and the polarization in the direction of translocation imposed by the specific sequences do not suffice to efficiently regulate the motor directionality.

To test whether stimulation of SpoIIIE motor activity by SRS could be responsible for the faster triplex displacement rate, we explored different values for *p*_*ATP*_., where *p*_*ATP*_^*SRS*^ ≠ *p*_*ATP*_^*NS*^. When increasing the *p*_*ATP*_^*SRS*^/*p*_*ATP*_^*NS*^ ratio (while fixing *p*_*ATP*_^*NS*^ = 0.012), *θ*_*SRS/NS*_ decreased non-linearly reaching the experimental range when *p*_*ATP*_^*SRS*^ > 0.4 (Fig. 5C). Indeed, values of *p*_*ATP*_^*SRS*^ above 0.4 did not further increase the triplex displacement rate, indicating that the number of proteins getting to SRS becomes eventually limited by the ATP-independent steps of the biochemical cycle of translocation (*i*.*e*., association, dissociation and diffusion). When using an equivalent probability (*p*_*dir*_) for SpoIIIE to translocate in any direction the model can satisfactorily reproduce the experimental results fulfilling the parsimony principle and revealing the central role of catalytic regulation of SpoIIIE by SRS in directionality regulation. Next, we sought to evaluate if coupling activation by SRS with orientation regulation could improve the model capacity to reproduce the SpoIIIE dynamics. Thus, we set the directionality parameter described previously to modulate the orientation of SpoIIIE with an intermediate efficiency (70% of recognition) and with 100% as control. The values of *p*_*ATP*_^*SRS*^ reproducing the experiments were lower than when no directionality was imposed, while the overall quality of the fittings remained comparable to the case in which no directionality bias was imposed by SRS. Interestingly, we observe that there is a very limited range of probabilities of activation by SRS in which the model could reproduce the experimental curves when the directionality parameter was introduced (Fig. S7). Thus, according to our modelling results, translocation directionality would not be regulated by the higher affinity of SpoIIIE for SRS but rather by the higher probability of activation of the ATP-fueled translocation activity of SpoIIIE upon encountering SRS. Favoring the activation of SpoIIIE whose orientation matches that of SRS or that are re-oriented by SRS may contribute to the directionality mechanism, but faithful achievement of correct directionality requires in all cases the motor activity modulation by SRS.

## Discussion

In this study, we investigated the detailed mechanisms by which SRS sequences regulate the SpoIIIE motor directionality by combining single-molecule imaging and fast kinetics translocation assays with mathematical modeling. Current models agree that specific sequences determine SpoIIIE directionality according to their orientation and that SpoIIIE ATPase activity and affinity are higher when SRS are present on DNA^6,11,12^. However, it remained unclear if these sequences are necessary or sufficient to trigger motor translocation and whether this is the main mechanism by which they regulate directionality.

The capacity of non-specific DNA to trigger the translocation activity of the SpoIIIE motor is a major source of discrepancy between previously proposed models for directionality regulation^11,12^. Our strategy employing atomic force microscopy imaging allowed the direct observation of SpoIIIE complexes after full translocation cycles on linearized DNA. This finding was further confirmed by fast kinetics translocation assays. These results strongly contradict previous models proposing that SpoIIIE or its homologue in *Escherichia coli* (FtsK) assemble into hexamers and begin translocation solely if specific sequences (SRS and KOPS for SpoIIIE and FtsK respectively) are present on DNA^12,18,19^. Our modelling results predict that the probability of activation upon interaction with non-specific DNA is ~1%. *In vitro* studies have shown that SpoIIIE/FtsK processivity ranges between 5 and 20 kb^9,16^ while in vivo SRS/KOPS sequences are spaced by ~12 kb^20^. It is then reasonable that motors maintain a certain activation probability when non-specifically bound, both to be able to continue translocating non-specific DNA after interaction with SRS/KOPS and to stochastically start translocating if the most proximal SRS/KOPS sequence in the chromosome is beyond the exploratory lengths determined by 1D diffusion (Fig. 5D ‘Active search pathway’).

To evaluate how specific sequences regulate the translocation activity and directionality of SpoIIIE, we introduced SRS repeats to the DNA substrates. Quantitative AFM imaging showed that the mean number of SpoIIIE reaching the DNA ends was independent of SRS, suggesting that the total number of translocating complexes is not increased by the presence of the specific sequences. Interestingly, single-round translocation measurements revealed that translocating SpoIIIE complexes reach the DNA end next to SRS faster than the opposite end of DNA. Therefore, SRS is, as expected, favouring the translocation of SpoIIIE towards a specific DNA end. However, two alternative molecular mechanisms could be responsible for regulating SpoIIIE translocation directionality: (i) either SRS recruit and accommodate the protein that will be ready to translocate in a specific direction upon ATP binding or (ii) SRS modifies the catalytic activity of the motor (*e*.*g*. modifying ATP accessibility) and thus creates a bias in the translocation directionality.

To discriminate between the two mechanisms, we expanded the mathematical model to reproduce the experimental triplex displacement traces. Simulations showed that the higher affinity of SpoIIIE for SRS coupled with 100% efficiency in protein orientation can not reproduce the translocation dynamics of SpoIIIE, strongly suggesting that directionality is not regulated by SpoIIIE being recruited and oriented by SRS. On the contrary, when SRS was allowed to modulate the translocation activity of SpoIIIE, the simulations recovered the experimentally observed kinetics when the probability of activation by SRS was ~30 times higher that by non-specific DNA. Then, combined with the lower dissociation probability from SRS (*p*_*off*_^*NS*^/*p*_*off*_^*SRS*^ ~ 2.3), our model predicts that SpoIIIE is ~70 times more likely to start translocating when bound to SRS than to non-specific DNA. Adding protein orientation by SRS improves the efficiency of the directionality regulation mechanism and allows to reach equivalent effects with lower probabilities of activation by SRS, but still this activation probabilities remain at least 10 times higher than when SpoIIIE is interacting with non-specific DNA. Altogether, our experimental and modeling results contrast with directionality regulation models proposed for SpoIIIE and its homologue FtsK^12,19^ while proving that SRS sequences do not only increase the ATPase activity of SpoIIIE as it has been previously shown^11,12^, but also have a determinant effect in the initiation of DNA translocation.

Rather than SpoIIIE being recruited and re-oriented by SRS, the mechanism defining directionality is exclusively dependent on the capacity of SRS to modulate the activation and translocation of SpoIIIE. Additionally the randomly occurring match between SRS and SpoIIIE orientation could finetune or reinforce synergically the SRS triggering action to safeguard translocation start in a non-favorable direction during sporulation. This *in vitro* observations can indeed account for the *in vivo* behavior of SpoIIIE if transport of DNA was occurring through a preexisting DNA-conducting pore before the final closure of the septum as it has been proposed previously^21,22^. As in the *in vivo* context the concentration of ATP will not be rate limiting in the protein catalytic cycle; this mechanism may have evolved to an optimal where directionality regulation relies on a rapid local search by 1D diffusion to bind stably to SRS and a robust catalytic modulation of the enzyme translocation activity if orientations match (Fig. 5D, ‘Polarity match pathway’).

Future *in vivo* studies with high spatial and temporal resolution should hold the key to further test the new directionality regulation model proposed in this work.

## Materials and Methods

### SpoIIIE purification and activity

SpoIIIE was expressed and purified as described previously^6^. ATPase measurements were performed in identical buffer and temperature conditions to those employed for incubation of the protein prior to AFM imaging and triplex displacement measurements with the addition of reagents from EnzCheck Pyrophosphate Assay Kit (Molecular Probes, USA). ATPase activity was measured as the initial velocity of release of Pi from ATP. Protein concentration was in all cases ~10 nM monomer. At this concentration SpoIIIE is mostly in hexameric state^11^ ensuring a stoichiometry of ~1 hexamer *per* DNA molecule.

### DNA substrates

DNA substrates were assembled by cloning duplex oligos containing 3-mer repeats of SRS (GAGAAGGGAGAAGGGAGAAGGG) or a random sequence replacing the SRS with equivalent CG content (GGAGGCGGGAGGCGGGAGGCGG) into the SpeI site of a derivative of the pBS SK(+) and the final products were verified by sequencing as described previously (Levy *et al*. 2005).

### AFM imaging and quantification

Mica (Goodfellow, France) was freshly cleaved and 1 μl of a 1 mM NiCl_2_ was deposited, incubated for 1 min and rinsed with 1 ml of deionized water (Millipore, Germany). Samples (2 nM DNA + 15 nM protein) were mixed and incubated for 2 minutes before deposition into the mica. Samples were deposited for 2 min and next rinsed with 3 ml of deionized water, dried with nitrogen and kept in a desiccator until imaging. AFM Images were obtained with a Nanoscope IIIa microscope (Veeco, France) equipped with a type-E scanner and operating in tapping mode in air using AC160 TS Olympus cantilevers (Olympus Corporation, Japan) as described^23^.

Image analysis was carried out using MATLAB (MathWorks, USA) and distributions of the localization of proteins on DNA were represented as relative frequency histograms with bin sizes of 50 nm. The relative frequency of SpoIIIE at SRS or DNA ends (*F*) was quantified as the ratio between the number of SpoIIIE located at the first bin $f({x}_{1})$ and the total number of proteins bound to DNA $\sum _{i=1}^{n}\,f({x}_{i})$:

1 $$F=\frac{f({x}_{1})}{{\sum }_{i=1}^{n}\,f({x}_{i})}$$

The absolute error of the relative frequency estimation for the first bin displayed in Fig. 1C and F was calculated from the error propagation of Eq. 1 assuming an uncertainty of ±1 SpoIIIE proteins *per* bin.

A detailed description of image analysis procedure and quantification is given in the Supplementary Information section.

### Triplex displacement assays

Fluorescent triplex substrates were prepared as described previously^6^ by using the same DNA substrates as for AFM imaging and a triplex forming oligonucleotide bearing a 5′-TAMRA fluorophore (Eurogentec, Belgium). Triplex displacement reactions were conducted at 27 °C following fluorescence anisotropy (FA) changes using a microplate reader, (excitation set to 530 nm and emission measured at 580 nm). 30 μL of 10 nM SpoIIIE was rapidly mixed with 30 μL of DNA-Triplex (1.2 nM) and FA signal was followed over time with a time resolution of ~7 s and a dead time of 15 s.

### Mathematical model encoding for SpoIIIE/DNA interactions

SpoIIIE-DNA dynamics were studied by means of a combined deterministic/non-deterministic mathematical model in which *m* DNA molecules were represented as single arrays containing *n* sites to which *r* SpoIIIE proteins can bind/unbind with probabilities *p*_*on*_*/p*_*off*_ respectively (Fig. 2). Bound SpoIIIE can unbind or slide (1D diffusion) along the DNA to another site a sliding distance *sld*_*m*_ ± *sld*_*s*_ (*mean* ± *sd*, in base pairs). All interactions not involving ATP-dependent mechanisms were modelled as a Markov process. In the presence of ATP, DNA-bound SpoIIIE can start translocating with a probability *p*_*ATP*_. When interacting with SRS SpoIIIE translocation direction is set with a probability *p*_*dir*_. Depending on the substrates characteristics (*i*.*e*., with or without SRS) SpoIIIE can interact with *n*_*SRS*_ or *n*_*NS*_ sites with distinct probabilities for all the previously described processes.

Once activated, SpoIIIE can randomly translocate in either direction. Translocation was deterministically modeled as a movement along the DNA with uniform velocity *v*_*trans*_. For the sake of simplicity, it is assumed that during translocation, SpoIIIE can dissociate from DNA with probability *p*_*off*_ for all types of sites. In case of collision between two SpoIIIE molecules only one will remain bound with translocating complexes taking priority over bound or diffusing SpoIIIE. When reaching the DNA end (by 1D diffusion or translocation), SpoIIIE proteins stop and can dissociate with a probability *p*_*off*_ or diffuse back to another site, reflecting on the boundaries. The relative frequency of SpoIIIE bound to SRS in the absence of ATP (for DNA_*SRS*_) was calculated as the temporal average of the ratio between the total number of proteins bound to SRS and the total number of proteins bound the DNA substrate. In the presence of ATP, the relative frequency of SpoIIIE arriving at the DNA ends (for DNA_*SRS*_ and DNA_*NS*_) was calculated as the temporal average of the ratio between the number of SpoIIIE reaching both DNA ends positions and the total number of proteins bounded to the DNA substrate. In all the cases, the temporal averages were calculated over the last 1,000 MCS (see Supplementary Information).

To simulate the triplex displacement kinetics, the presence of a triplex forming oligonucleotide on the DNA end next to the SRS sequence (which is replaced by non-specific base pairs in the DNA_NS_ substrates) was added. When SpoIIIE, by translocation, arrives to the DNA end, it releases the triplex with probability *p*_*triplex*_ = 1 and this DNA molecule is no longer considered during the remainder of the simulation. To quantitatively compare the simulation results to experimental data it was assumed that the accumulated number of released triplex in the simulations is linearly proportional to a theoretical anisotropy signal and the ratio between the area under the curves of kinetic traces for DNA_*SRS*_ and DNA_*NS*_ was calculated (see Supplementary Information). Additional model details, parameterization and quantification are given in the Supplementary Information.

## Electronic supplementary material

Supplementary Information

Movie_M1

Movie_M2

Movie_M3

Movie_M4

Movie_M5
