# Supplementary material for: Sequence-dependent catalytic regulation of the SpoIIIE motor activity ensures directionality of DNA translocation
Source: Sci Rep. 2018 Mar 27;8:5254. doi: 10.1038/s41598-018-23400-8 (PMC5869595; doi:10.1038/s41598-018-23400-8)
Supplement: Supplementary file 1 — Supplementary Information [file 41598_2018_23400_MOESM1_ESM.pdf]

Supplementary Information for:

Sequence-dependent catalytic regulation of the SpoIIIE motor activity ensures directionality of DNA translocation

Osvaldo Chara, Augusto Borges, Pierre-Emmanuel Milhiet, Marcelo Nöllmann and Diego I. Cattoni

Content

Figure S1. Distribution of SpoIIIE in DNA with and without SRS in presence and absence of ATP and ATPase activity measurements.

Figure S2. Anomalous diffusion is the main factor localizing SpoIIIE to SRS in the absence of ATP

2.1 Discussion on the robustness of the mathematical model for SpoIIIE interactions with DNA in absence of ATP and mechanistic interpretation of the results.

2.1.1 The rate at which SpoIIIE finds its target sequence is proportional to the one-dimensional exploring lengths (Fig. S2)

2.1.2 Pon_{SRS} is not the main factor localizing SpoIIIE to SRS (Fig. 3B).

2.1.3 $Poff_{SRS}$ coupled to anomalous diffusion localizes SpoIIIE to SRS (Fig. 3C)

Figure S3. Increasing translocation velocity or probability of activation by ATP enhances SpoIIIE localization to the DNA ends for both DNA_{NS} and DNA_{SRS} substrates

Figure S4. Kinetics of SpoIIIE redistribution to SRS and DNA ends

Figure S5. Residence, exploration and translocation times of SpoIIIE when interacting with DNASRS

Figure S6. Effect of the presence of triplex in AFM distributions

Figure S7. Modulation of directionality of SpoIIIE by SRS as function of activation probability by ATP.

Table S1. Equilibrium binding and kinetic constants previously obtained for DNA_{NS} and DNA_{SRS}

Movies M1, M2 and M4. Real-time dynamics of SpoIIIE interaction with DNA_{SRS} in absence of ATP and DNA_{NS} and DNA_{SRS} in the presence of ATP

Movie M3. Single molecule dynamics of SpoIIIE interaction with DNA_{SRS} in the presence of ATP

Movie M5. Triplex displacement kinetics: concentration mechanism and catalytic mechanism

Supplementary Material and Methods

AFM image analysis and quantification

Modeling

Model parameterization and simulation details

Quantification of model-predicted SpoIIIE distributions in presence and absence of ATP.

Quantification of model-predicted triplex displacement induced by SpoIIIE in the presence of ATP.

Quantification of model-predicted protein residence time along DNA substrates.

References

Figure S1. Distribution of SpoIIIE in DNA with and without SRS in presence and absence of ATP and ATPase activity measurements.

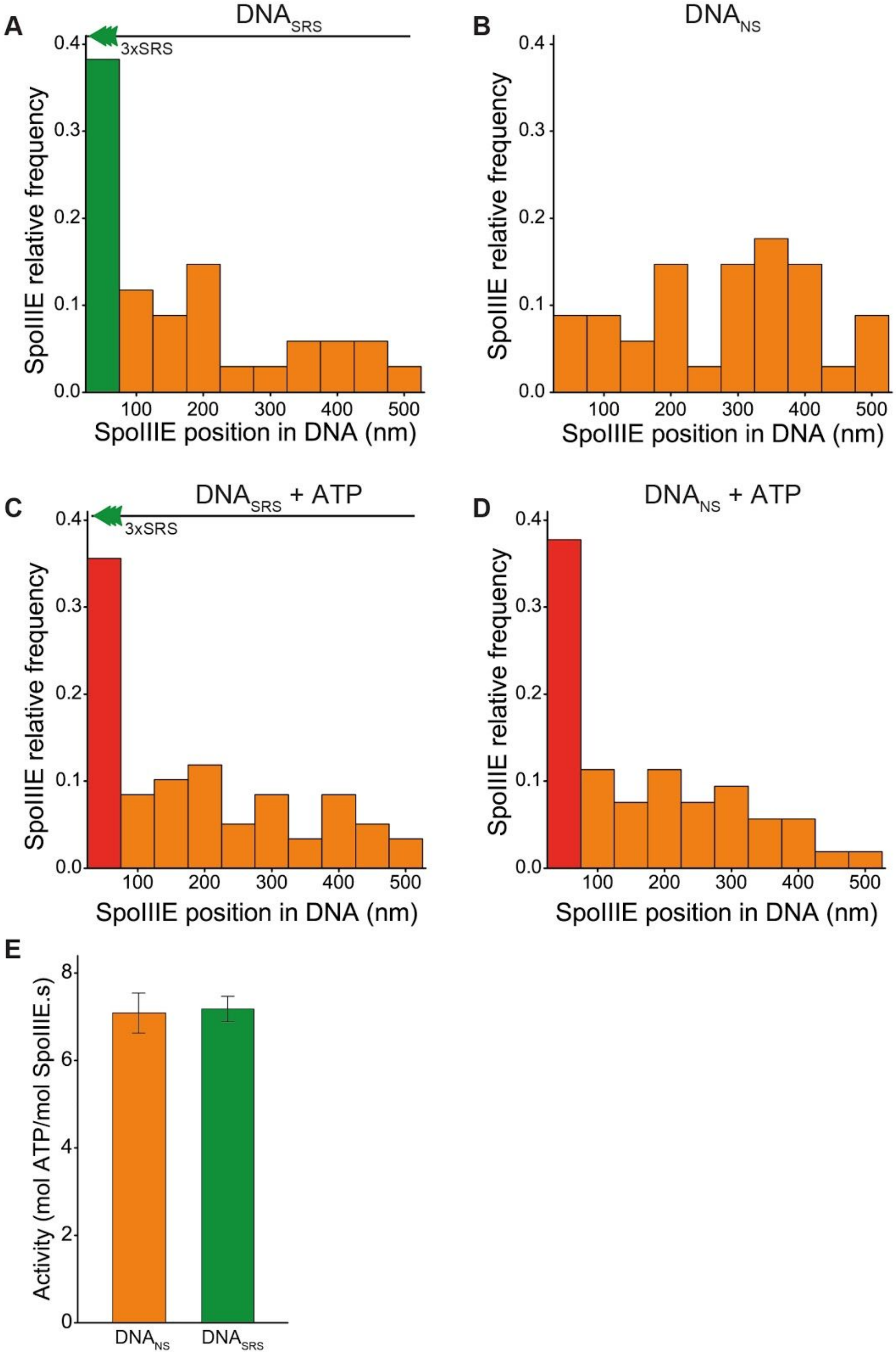

(A-D) Histograms of SpoIIIE distribution in DNA with and without SRS (DNA_{SRS} and DNA_{NS} respectively) in absence (**A-B**) and presence of ATP (**C-D**). Upper scheme in panels A and C indicates the localization of SRS sequences in DNA_{SRS}. Orange and green columns in panels A and B indicate bins containing non-specific and SRS sequences respectively. Red columns in panels C and D indicate DNA ends reached by SpoIIIE by active translocation. Panels A-B reprinted with permission from EMBO Reports [1]. 30 to 40 molecules from three independent biological replicates were analyzed in each condition.

(E) ATPase activity of SpoIIIE in AFM and triplex conditions. 10 nM of SpoIIIE were mixed with 2nM DNA substrates employed for AFM and triplex displacement measurements (DNA_{NS} and DNA_{SRS}) and specific ATPase activity was measured as the initial rate of inorganic phosphate (Pi) release in the linear region of the absorbance curve at 360 nm at 27°C. SpoIIIE had equivalent specific ATPase activity for both substrates and similar values to what it has been previously reported (~ 10 mol ATP/s/mol SpoIIIE, [1,2]).

Figure S2. Anomalous diffusion is the main factor localizing SpoIIIE to SRS in the absence of ATP

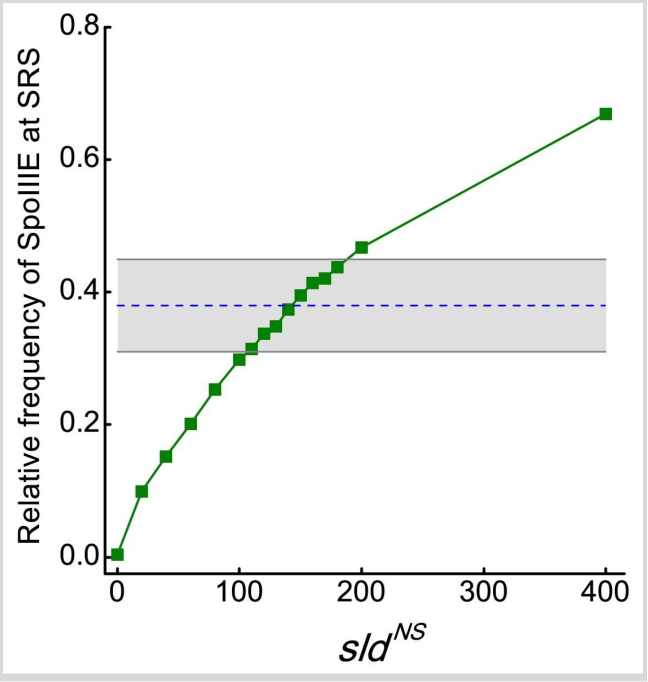

Model-predicted relative frequency of SpoIIIE at SRS sequences in the absence of ATP evaluated as function of sliding length (sld^{NS}) of SpoIIIE within the non-specific DNA sequences. Solid lines connecting symbols are only a guide to the eye.

2.1 Discussion on the robustness of the mathematical model for SpoIIIE interactions with DNA in absence of ATP and mechanistic interpretation of the results.

The results depicted in Figure 3A-C and S2 were obtained on the basis of a previously developed mathematical model, capable of reproducing experimental observations from distributions of SpoIIIE in the absence of ATP on linearized DNA having SRS (evaluated as the relative frequency of SpoIIIE proteins interacting with SRS, F_{SRS} = 0.38 ± 0.07). The parameters values employed in the simulations were based on experimental results (Table S1). To further evaluate the robustness of the model we decided to explore a wider region of the parameters space regarding

binding/unbinding and diffusion of SpoIIIE along DNA having SRS and non-specific sequences (Fig. 2B i of the main text):

2.1.1 The rate at which SpoIIIE finds its target sequence is proportional to the one-dimensional exploring lengths (Fig. S2)

We quantitatively tested the effect of anomalous diffusion in the efficiency of the previously proposed target search mechanism by varying the sliding length in the non-specific DNA region (sld^{NS}) from 1 to 400 bp whilst the remaining parameters were fixed at the previously optimized values (see Table 1). As expected, the rate to find SRS (unpublished data) and the amount of SpoIIIE bound to SRS at the steady state increased proportionally to the exploration distances defined by the sliding length on non-specific DNA (Fig. S2). On the contrary, in the absence of anomalous diffusion (sld^{SRS} = sld^{NS} = 145 bp) the redistribution of SpoIIIE to SRS was no longer observed (unpublished data).

The sliding lengths in the simulations that best described the previous experimental data (145 bp ~ 50 nm) are consistent with previously experimentally measured sliding lengths [3–6]. The difference in sliding lengths between SRS and non-specific sequences from our simulations (~100 fold) is consistent with previous studies showing that small differences in interaction energies between non-specific and specific DNA-protein complexes (1-2 kBT) can lead to differences in sliding length of ~10-100 folds. It has been proposed that these differences in sliding lengths originate from conformational changes in the protein structure when binding to specific sites [7]. These predictions are consistent with SpoIIIE going through conformational and/or oligomerization state changes upon binding to SRS as it has been previously suggested [1]. Note that in our simulations the sliding lengths values are normally distributed with a standard deviation of 25% from the mean value. In a previous work the best fitting sliding length for equivalent experimental data was 90 ± 22 bp [1] whereas in our current modelling strategy the best fitting value is 145 ± 36 bp. This new values overlap with previous findings within one sigma while yielding much lower error margin.

2.1.2 P_{on}^{SRS} is not the main factor localizing SpoIIIE to SRS (Fig. 3B).

In a previous study [1], from pre-equilibrium binding curves, it has been shown that SpoIIIE association probability is independent of DNA sequences (Table S1) and thus in the model implementation it was assumed that $p_{on}^{SRS} = p_{on}^{NS}$ (Table 1). However, competing models proposed that the SpoIIIE homolog in *E. coli* FtsK, assembles into hexamers by binding to specific sequences solely through a 3D diffusion mechanism (*i.e.*, preferential loading model [8,9]). This model implies that SpoIIIE/FtsK type of motors can not explore DNA by 1D diffusion and that the association rate is governing binding of SpoIIIE/FtsK to SRS/KOPS. We explored p_{on}^{SRS} two orders of magnitude above and below the values that reproduced previous experimental findings while varying the diffusion properties of SpoIIIE. When 1D diffusion was homogeneous (*i.e.* $sld^{SRS} = sld^{NS}$) the experimental distributions of SpoIIIE could not be recovered (orange dots in Fig.3B). Whereas when diffusion was absent, the simulations reproduced the previous distribution of SpoIIIE along DNA only when $p_{on}^{SRS} = 1$ (grey triangles in Fig. 3B). An association probability value of 1 has limited physical meaning since it implies that every protein colliding with the DNA will bind with an efficiency of 100%, whereas even proteins with the highest affinity binding for DNA don't show this property. Increasing p_{on}^{SRS} when anomalous diffusion allowed to reproduce the experimentally obtained F_{SRS} values for all the p_{on}^{SRS} range evaluated (green squares in Fig. 3B). Overall, and in strong contradiction with previous models, these results suggest that, even for a large range of parameter values, p_{on}^{SRS} does not have a substantial effect on the protein localization into SRS.

2.1.3 P_{off}^{SRS} coupled to anomalous diffusion localizes SpoIIIE to SRS (Fig. 3C)

According to previous experimental results the affinity of SpoIIIE for specific sequences is governed by the dissociation process (see Table S1), thus we employed our mathematical model to further evaluate the role of the dissociation probability from SRS sequences in the distribution of SpoIIIE in DNA (*i.e.*, varying the ratio between p_{off}^{SRS} and p_{off}^{NS}).

In the absence of anomalous diffusion (either when $sld^{SRS} = sld^{NS} = 145$ bp or when $sld^{SRS} = sld^{NS} = 0$ bp), SpoIIIE was nearly not found at SRS for all the p_{off}^{SRS} values

tested (orange dots and grey triangles in Fig. 3C). This suggest that the anomalous diffusion is probably the most important mechanism contributing to the experimentally observed SpoIIIE localization at SRS. In agreement with this, when including anomalous diffusion in our simulations, for SRS dissociation probabilities equal or 4 times higher than dissociation from non-specific DNA, we still observed localization of SpoIIIE into SRS (compare results depicted in figure 3C when p_{off}^{SRS} = 0.006 and p_{off}^{SRS} equals 0.014 and 0.022). This findings are in agreement with previous equilibrium binding measurements where the dissociation probability from SRS plays a role in the localization of SpoIIIE to SRS. The stable interaction between SpoIIIE and SRS combined with asymmetric diffusion (see below) are key players in the protein localization and binding to specific sequences. These results are not surprising considering that the main driving force for protein/DNA association are non-specific electrostatic interactions, while additional contacts and possibly conformational changes once the protein is bound to SRS would stabilize the SpoIIIE hexamer and define a slower dissociation rate compared to non-specific DNA.

Figure S3. Increasing translocation velocity or probability of activation by ATP enhances SpoIIIE localization to the DNA ends for both DNA_{NS} and DNA_{SRS} substrates

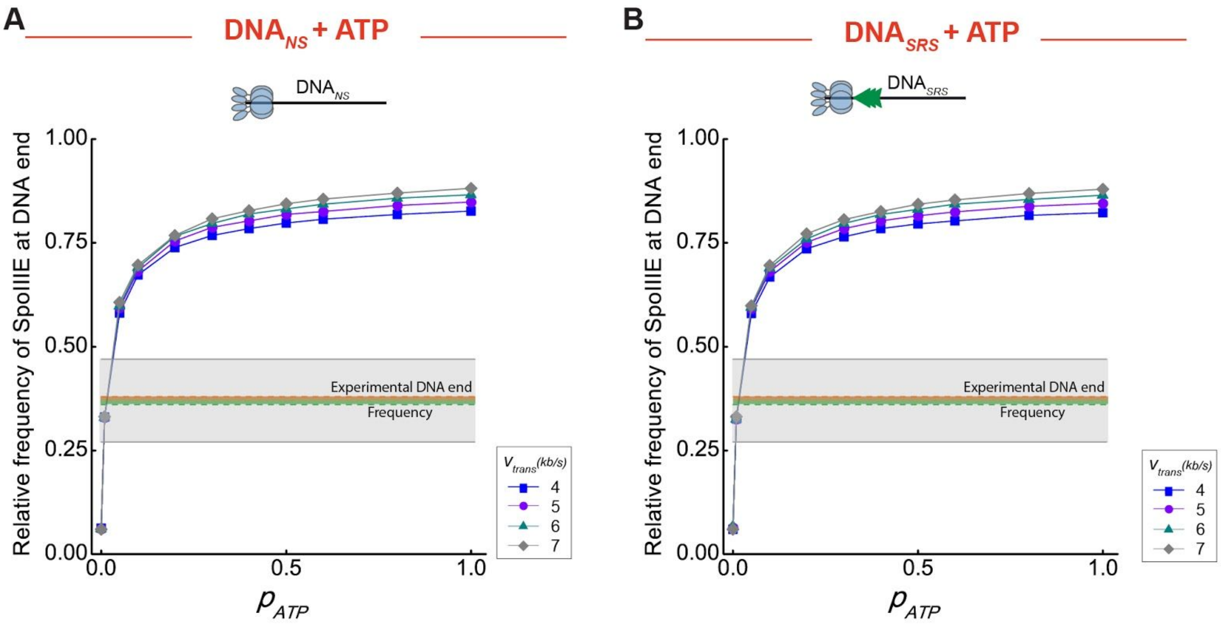

(**A-B**) Model-predicted relative frequency of SpoIIIE reaching DNA ends for substrates without (**A,** DNA_{NS}) and with SRS (**B,** DNA_{SRS}) in the presence of ATP as a function of ATP activation probability (p_{ATP}) for different translocation velocities (v_{trans}). Green-orange gradient and grey shadow area indicates the experimental mean relative frequency and standard deviation for both substrates in the presence of ATP. All other parameter values were kept at the values depicted in Table 1. Upper scheme represents SpoIIIE reaching the DNA ends for substrates with or without SRS. In all simulations the relative frequency of SpoIIIE reaching DNA end was estimated once simulations reached steady state conditions. Solid lines connecting symbols are only a guide to the eye.

Figure S4. Kinetics of SpoIIIE redistribution to SRS and DNA ends

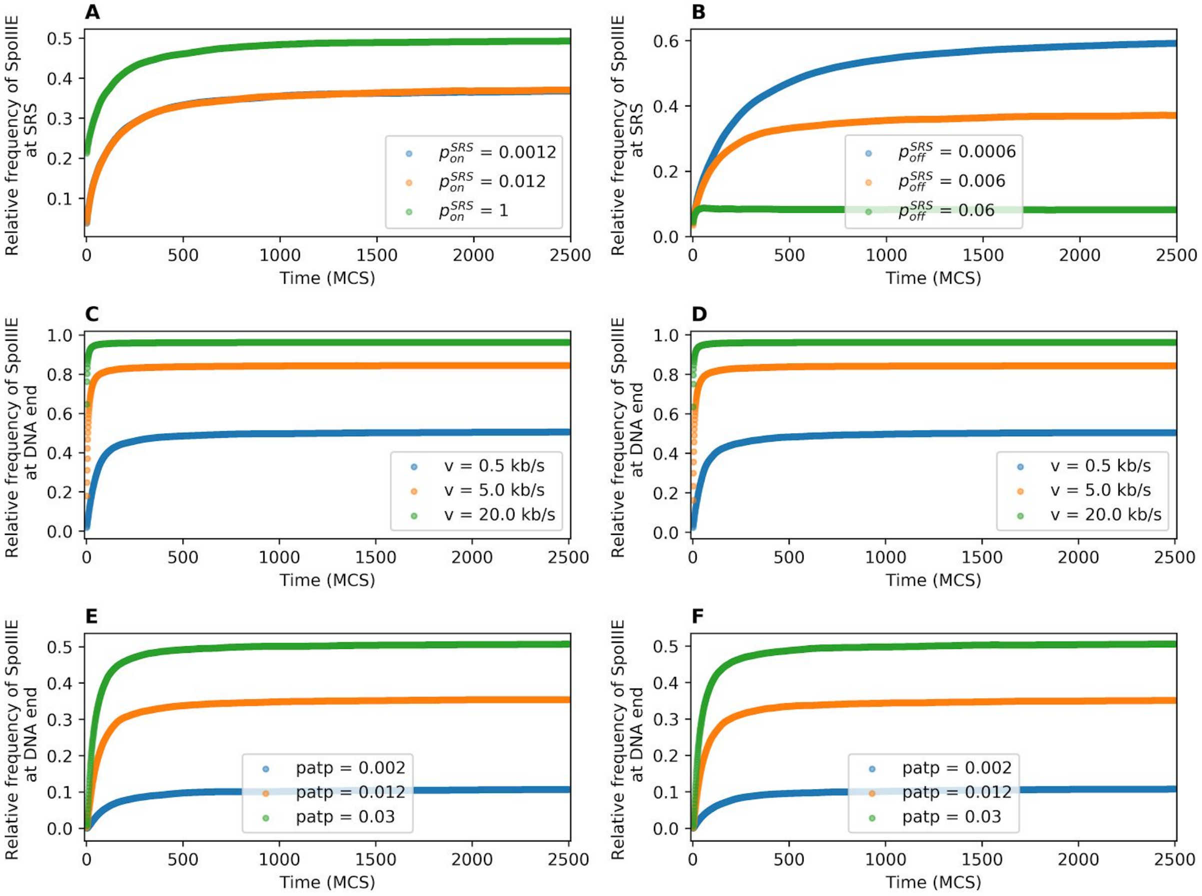

Kinetics of SpoIIIE redistribution to SRS sites or to the DNA ends for the different parameters values from figures of the main text. Kinetic traces in each panel were obtained by fixing the remainder of the parameters to the values described in Table 1 and by varying the parameter indicated in the inset. Panels A and B depict the kinetic traces of DNA_{SRS} in the absence of ATP for different values of $p_{on}{}^{SRS}$ and $p_{off}{}^{SRS}$, respectively. Panels **A** and **B** parameters correspond to Figure 3 panels B and C, respectively. Vertical axis represent the relative frequency of SpoIIIE at SRS. Panels C and E depict the kinetic traces of DNA_{NS} **(C)** and DNA_{SRS} **(D)** in the presence of ATP for different values of translocating velocity (v_{trans}) shown in the inset, with activation probability p_{ATP} = 1. Panels D and F depict the kinetic traces of DNA_{NS} **(E)** and DNA_{SRS} **(F)** in the presence of ATP for different values of activation probability (p_{ATP}) and assuming v_{trans} = 5 kb/s. Panels C and D parameters correspond to figure 4 panel B, and

panels E and F to figure 4 panel C. Horizontal axis represent time evolution in Monte Carlo steps (MCS). Vertical axis represent the relative frequency of SpoIIIE at DNA end.

Figure S5. Residence, exploration and translocation times of SpoIIIE when interacting with DNA_{SRS}

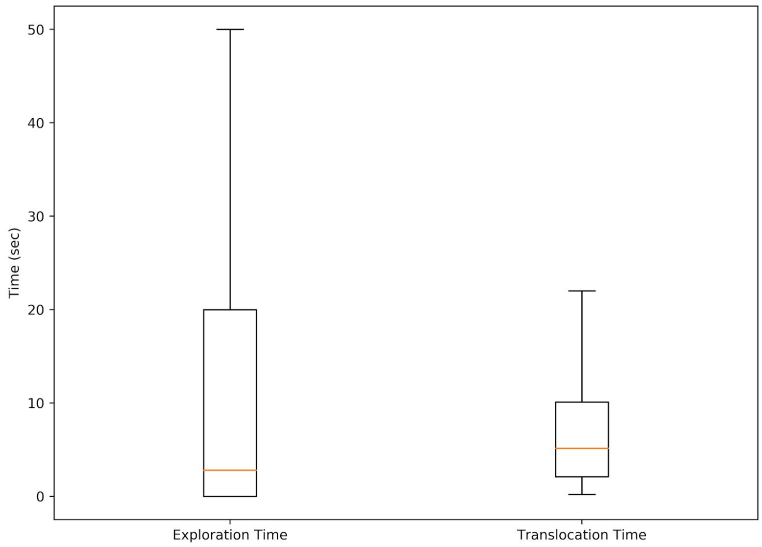

Boxplots of the SpoIIIE exploration time to reach SRS (left, 2.8 ± 24 s) and time SpoIIIE remains bound to DNA once it starts to translocate (right, 5.2 ± 6.8 s). Values between parentheses represent median and standard deviation. The activation probability was set to $p_{ATP}^{NS} = p_{ATP}^{SRS} = 0.012$, the rest of the parameters were set at the values depicted in Table 1. Each boxplot consist of 10,000 individual simulations of 2,000 Monte Carlo steps each.

Our modelling strategy assumes that *in vitro* SpoIIIE binds to DNA and explores it by 1D diffusion. However, *in vivo* SpoIIIE is bound by a linker to the sporulation septum. Two hypotheses may be confronted to account for the exploration of DNA by SpoIIIE during sporulation. On the one hand, if the linker is assumed to be extremely rigid, the natural movement of the DNA confined within the closing septum could provide an exploratory mechanism by which SpoIIIE finds SRS. On the other hand, if the linker is assumed as a flexible structure, the natural movement of DNA combined with the movement of SpoIIIE would act synergistically amplifying the exploratories lengths and thus diminishing the exploratory times. Considering that the linker is putatively unstructured [2] and given its inherent length it seems unlikely that the linker could be considered as a rigid structure. Moreover, previous experiments have

suggested that the linker can be stretched to lengths of over ~ 40 nm [10]. Future *in vivo* experiments tracking simultaneously, with high temporal and spatial resolution, SpoIIIE and DNA will allow to refine this mechanistic details of SRS fiding by SpoIIIE.

Figure S6. Effect of the presence of triplex in AFM distributions

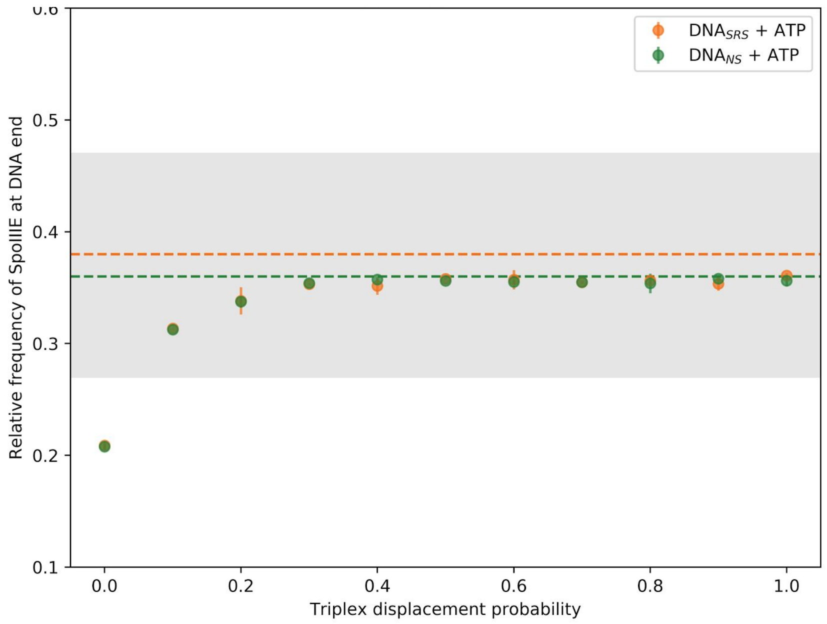

Model-predicted relative frequency of SpoIIIE reaching DNA ends for DNA_{SRS} (green circles) and DNA_{NS} (orange circles) as a function of the triplex displacement probability with p_{atp}=0.012. The number of SpoIIIE located at both DNA ends was quantified as described in Material and Methods. The probability of SpoIIIE to displace triplexes was varied from 0 (SpoIIIE can not displace the triplex in any case and thus it dissociates from DNA) to 1 (SpoIIIE displaces the triplex a 100% of the time). Orange and green dashed lines and grey shadow area indicate the experimental mean relative frequency and standard deviation respectively for both DNA substrates in the presence of ATP. Error bars corresponds to standard deviation from 4 simulations employing 500 DNA molecules and 1000 SpoIIIE proteins. All other parameter values were set at the values depicted in Table 1.

Figure S7. Modulation of directionality of SpoIIIE by SRS as function of activation probability by ATP.

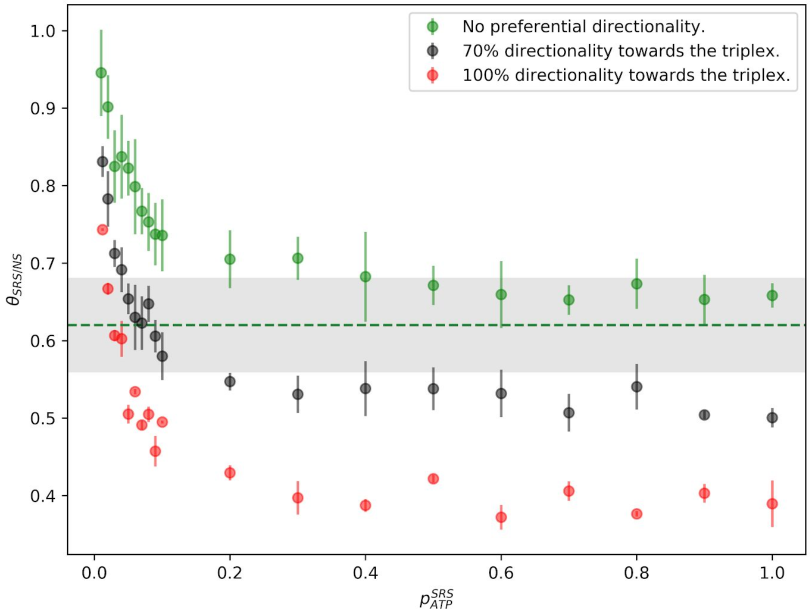

Effect of SRS in triplex displacement rates quantified as the ratio between the area under the kinetic traces for DNA_{SRS} and DNA_{NS} ($\Theta_{SRS/NS}$). Circles and error bars indicate mean and standard deviation of the simulation results by varying ${p_{ATP}}^{SRS}$ while maintaining ${p_{ATP}}^{NS}$ constant at 0.012. Green data represent the case of figure 5 panel C, *i.e.*, p_{dir} = 0.5. Black and red dots correspond to p_{dir} = 0.7 and p_{dir} = 1, respectively. Grey shaded area indicates the mean and standard deviation of experimental $\Theta_{SRS/NS}$.

Table S1. Equilibrium binding and kinetic constants previously obtained for DNA_{NS} and DNA_{SRS}

**Substrate**	K_D (nM)	k_{on}^{app} (s^{-1})	k_{off} (s^{-1})	$k_{off\text{-}ATP}$ (s^{-1})
DNA_{NS}	61 ± 12	0.23 ± 0.04	0.14 ± 0.02	0.15 ± 0.01
DNA_{SRS}	22 ± 4	0.21 ± 0.03	0.06 ± 0.01	0.07 ± 0.01

Reprinted from [1] with permission of EMBO reports.

Movies M1, M2 and M4. Real-time dynamics of SpoIIIE interaction with DNA_{SRS} in absence of ATP and DNA_{NS} and DNA_{SRS} in the presence of ATP

A. Instantaneous localization of SpoIIIE in DNA_{SRS} without ATP (**M1**) and DNA_{NS} and DNA_{SRS} substrates with ATP (**M2** and **M4** respectively) for 5 representative DNAs. Individual interaction events (binding, diffusing and, when ATP is present, translocation) of SpoIIIE with DNA in real-time are depicted with green solid lines.

B. Cumulative distributions of SpoIIIE in DNA_{SRS} without ATP (**M1**) and DNA_{NS} and DNA_{SRS} with ATP (**M2** and **M4**). The cumulated number of SpoIIIE in real-time at each DNA coordinate from panel A was color-coded according to the scale depicted on the right.

For all movies, in A and B ordinate and abscissa indicate the index number of each simulated DNA and its corresponding coordinates in base pair (bp), respectively. For clarity reasons panel B in **M1** is zoomed at the 400 coordinates adjacent to the left DNA end containing the SRS sequence and in **M2 and M4** are zoomed at the first 200 coordinates adjacent to the left and right DNA ends to highlight the cumulative arrival of SpoIIIE at DNA ends.

Note at the end of the simulation in M1 the contrast between the dark green at 44 to 59 coordinates depicting the higher frequency of interaction of SpoIIIE with SRS and the deem green shadow distributed homogeneously in the remainder of DNA indicating non-specifically bound complexes. Similar observations can be made for M2 and M3 for the proteins accumulating at 1 and 2973 coordinates.

D. Kinetics of SpoIIIE localization to SRS (**M1**) and arrival to DNA ends (**M2** and **M4**). The relative frequency of SpoIIIE reaching the DNA ends (either left or right) was calculated from bin 1 of panel C and depicted as a function of the simulation time (Monte Carlo steps, MCS).

Simulation running time is indicated on the right central part of the image (2000 Monte Carlo steps total). 10 SpoIIIE and 5 DNA molecules were simulated simultaneously (see Figures 2 and S1, Material and Methods and Extended Material and methods for additional details). Parameter values for simulations are depicted in Table 1. For movie M3 $p_{ATP}^{SRS} = p_{ATP}^{NS} = 0.012$.

Movie M3. Single molecule dynamics of SpoIIIE interaction with DNA_{SRS} in the presence of ATP

Instantaneous localization of SpoIIIE in DNA_{SRS} in presence of ATP. The simulations were performed with a single DNA_{SRS} and single SpoIIIE complex. The parameter values were the same as in M4 (see above). Each of the three panels represent a region of the simulated DNA. The *x* axis represents the position along the DNA, the color code depicted on the right indicates the state of the binding site and the protein state (whether the protein is translocating or not) when interacting with that site. Blue shadowed region indicates the position of the SRS region.

Movie M5. Triplex displacement kinetics: concentration mechanism and catalytic mechanism

A-B. Model simulated kinetics of triplex displacement for SpoIIIE translocating in S_{NS} (orange dots) and S_{SRS} (green dots) scenarios when (**A**) $p_{ATP}^{SRS} = p_{ATP}^{NS} = 0.012$ and (**B**) $p_{ATP}^{NS} = 0.012$ and $p_{ATP}^{SRS} = 0.8$. Note that a higher affinity for SRS does not allow to reproduce the fastest displacement rate experimentally observed for substrates containing SRS whereas when a higher probability of activation by SRS is included in the model, triplex displacement occurs faster in DNA_{SRS} than in DNA_{NS} (see figure 5C of the main text and Table 1 for parameter values employed in this simulations).

Supplementary Material and Methods

AFM image analysis and quantification

Topographical images obtained by AFM (raw images after a 1st order, line wise flattening using the Nanoscope analysis software) were imported into MATLAB. A semi-automatic tracking algorithm allowed the operator to indicate the location of the DNA. The background of the imported images was binarized using as a threshold value that enhanced the contrast between DNA and the mica surface. Tracing of DNA was performed by using a maximum Hessian approach; protein was detected automatically by thresholding and localized by fitting with a moment centroid-determination routine.

AFM spatial distributions of SpoIIIE on the DNA substrates containing or not the SRS sequences in the presence and absence of ATP were obtained as follows: considering the resolution of AFM imaging we took a conservative approach and define a binning size of 50 nm when evaluating SpoIIIE distributions in DNA. This binning size is equivalent to ~147 bp, therefore the first bin from one of the extremities of DNA includes the SRS region. Since in our imaging conditions, for DNA_{SRS} we had no spatial reference indicating in which extremity are located the SRS sequences, we measured the distribution of SpoIIIE taking the central part of the DNA as the coordinate 500 nm and measuring the distance at which each SpoIIIE complexes were detected from the center as decreasing absolute distances. In this way, the total DNA length becomes 500 nm (starting from 0 at the DNA extremities) divided in 10 bins of 50 nm. SpoIIIE AFM distributions were calculated as the relative frequencies in each bin defined as the number of proteins bound at each location (bin) respect to the total number of proteins quantified for all DNAs. Error bars displayed in Figure 1 C and F of the main text were calculated as the absolute error for bin 1 obtained from the propagation of Equation 1 assuming an uncertainty of ±1 SpoIIIE proteins *per* bin.

Modeling

Model parameterization and simulation details

The model was evaluated in two *in silico* scenarios: 1) n DNA molecules having n_{SRS} specific SRS sequences surrounded by n_{NS} non-specific nucleotide bases (scenario

DNA_{SRS}, Fig. 2B) and 2) *n* DNA molecules having only n_{NS} non-specific bases (scenario DNA_{NS}, Fig. 2A). For the DNA_{NS} scenario, the 15 base pairs of the SRS sequences were replaced by non-specific base pairs. The model was parametrized such that simulations can be compared with previous [1] and here reported experimental results of AFM and triplex displacement assays (see main text). The number of non-specific binding sites in DNA_{NS} scenario (n_{NS}) was calculated as the total number of base pairs (N_{BP}= 2973) minus the estimated footprint of the SpoIIIE motor ($N_{SpoIIIE}$=17bp, based on the homology model from the crystallographic structure of FtsK, PDB:2IUU [11]). Finally, we considered the structure of SpoIIIEαβγ to calculate the number of specific sites in DNA_{SRS} (n_{SRS}). For each SRS sequence in DNA_{SRS}, SpoIIIE-γ can bind specifically to SRS while the motor (SpoIIIE-αβ) can bind up to three possible neighbouring sequences (due to the extension of the ~5 amino-acid long linker joining the γ- and β-domains). Thus, since in the DNA_{SRS} substrates there are three SRS sites, in the DMA_{SRS} scenario (*i.e.* simulated substrates) there will be 15 specific binding sites. Hence, for the DNA substrates employed in our studies, total *n* = 2,973 with n_{SRS} = 15 and n_{NS} = 2,958 base pairs, for the DNA_{SRS} scenario and n_{NS} = 2,973 for the DNA_{NS}.

Unless other conditions were specified, *m* and *r*, the numbers of DNA and SpoIIIE molecules were fixed in 500 and 1000, respectively maintaining a ratio [SpoIIIE]/[DNA] = 2/1, emulating our experimental conditions (AFM and triplex displacements).

To estimate the model transition probabilities (p_{on}^{SRS}, p_{off}^{SRS}, p_{on}^{NS}, p_{off}^{NS}, Table 1) we empirically related the experimentally measured association and dissociation rates obtained from pre-equilibrium binding measurements of SpoIIIE and short DNA containing or not SRS (Table S1, [1]) to the MCS probabilities assuming a simple two-state process (bound-unbound SpoIIIE):

$$k_{off}^{NS} = \frac{p_{off}^{NS}}{\Delta t} \text{ and } k_{off}^{SRS} = \frac{p_{off}^{SRS}}{\Delta t} \qquad \text{(Eq. S1)}$$

and

$$k_{on,app}^{SRS} = k_{on,app}^{NS} = \frac{p_{on}^{SRS}}{\Delta t} = \frac{p_{on}^{NS}}{\Delta t} \qquad \text{(Eq. S2)}$$

where *Δt* is the duration of a simulation step in the Monte Carlo simulations; k_{off}^{SRS} and k_{off}^{NS} represent the dissociation rates obtained from pre-equilibrium measurements

(concentration independent); finally, $k_{on,app}^{SRS}$ and $k_{on,app}^{NS}$ represent the apparent kinetic rate constants for binding estimated from our pre-equilibrium rate constants for DNAs having or not the SRS sequence, respectively (Table S1). k_{off}^{NS} was unaltered by the presence of ATP, thus for the sake of simplicity, for translocating SpoIIIE complexes the probability of dissociation was also set to p_{off}^{NS}. The best sliding lengths values (Fig. S2 and Table 1) describing the AFM data were estimated by systematically varying the sld^{NS}/sld^{SRS} ratio in our model, while keeping the parameter values obtained from equations S1 and S2 constant, until reproducing previous experimental data obtained in the presence of the specific SRS sequence by means of atomic force microscopy [1]. Δt in the simulations corresponds to 0.1 s.

Quantification of model-predicted SpoIIIE distributions in presence and absence of ATP. To contrast simulation and experimental results in the absence of ATP, the relative frequency of SpoIIIE bound at SRS was calculated as the temporal average of the ratio between the total number of proteins bound to the 15 base pairs representing the SRS sequence (45 to 59) and the total number of proteins bound along the substrate. In the presence of ATP, the relative frequency of SpoIIIE arriving at the DNA ends was calculated as the temporal average of the ratio between the number of SpoIIIE reaching both DNA ends positions (1 and 2973) and the total number of proteins distributed in the substrate. In all the cases, the temporal averages were calculated over the last 1,000 MCS of the simulations.

The model was implemented in a FORTRAN 90 code and it is available upon request.

Quantification of model-predicted triplex displacement induced by SpoIIIE in the presence of ATP.

The differences between triplex displacement simulations for the DNA_{SRS} and DNA_{NS} scenarios were quantified as the ratio ($\Theta_{SRS/NS}$) between the area under the kinetic trace in the presence and the absence of SRS as:

$$\Theta_{SRS/NS} = \frac{\int_0^{t_f} A_{SRS}(t)\, dt}{\int_0^{t_f} A_{NS}(t)\, dt} \quad \text{(Eq. S3)}$$

Where t represents time and $A_{SRS}(t)$ and $A_{NS}(t)$ represent the time-dependent fluorescence anisotropy signal (either experimentally obtained or from simulations) for SRS and non-specific substrates.

Quantification of model-predicted protein residence time along DNA substrates.

One *in silico* DNA substrate of 2974 base pairs, with an SRS sequence between bases 45 and 59 was incubated with a single SpoIIIE protein and 10,000 simulations were run for 2000 Monte Carlo steps each. The Exploration time was calculated as the time period between a binding event and the first time the protein reaches the SRS sequences. The translocation time was calculated as the time period between the protein activation and its dissociation from the DNA.

References

1. Cattoni, D. I. *et al.* SpoIIIE mechanism of directional translocation involves target search coupled to sequence-dependent motor stimulation. *EMBO Rep.* **14,** 473–479 (2013).
2. Ptacin, J. L. *et al.* Sequence-directed DNA export guides chromosome translocation during sporulation in Bacillus subtilis. *Nat. Struct. Mol. Biol.* **15,** 485–493 (2008).
3. Wang, Y. M., Austin, R. H. & Cox, E. C. Single molecule measurements of repressor protein 1D diffusion on DNA. *Phys. Rev. Lett.* **97,** 048302 (2006).
4. Farge, G. *et al.* Protein sliding and DNA denaturation are essential for DNA organization by human mitochondrial transcription factor A. *Nat. Commun.* **3,** 1013 (2012).
5. Hammar, P. *et al.* The lac repressor displays facilitated diffusion in living cells. *Science* **336,** 1595–1598 (2012).
6. Marklund, E. G. *et al.* Transcription-factor binding and sliding on DNA studied using micro- and macroscopic models. *Proc. Natl. Acad. Sci. U. S. A.* **110,** 19796–19801 (2013).
7. Slutsky, M. & Mirny, L. A. Kinetics of protein-DNA interaction: facilitated target location in sequence-dependent potential. *Biophys. J.* **87,** 4021–4035 (2004).
8. Bigot, S., Saleh, O. A., Cornet, F., Allemand, J.-F. & Barre, F.-X. Oriented loading of FtsK on KOPS. *Nat. Struct. Mol. Biol.* **13,** 1026–1028 (2006).
9. Graham, J. E., Sherratt, D. J. & Szczelkun, M. D. Sequence-specific assembly of FtsK hexamers establishes directional translocation on DNA. *Proc. Natl. Acad. Sci. U. S. A.* **107,** 20263–20268 (2010).
10. Fiche, J.-B. *et al.* Recruitment, assembly, and molecular architecture of the SpoIIIE DNA pump revealed by superresolution microscopy. *PLoS Biol.* **11,** e1001557 (2013).
11. Massey, T. H., Mercogliano, C. P., Yates, J., Sherratt, D. J. & Löwe, J.

Double-stranded DNA translocation: structure and mechanism of hexameric FtsK. *Mol. Cell* **23,** 457–469 (2006).
